# Impact factor, H index, peer comparisons, and *Retrovirology*: is it time to individualize citation metrics?

**DOI:** 10.1186/1742-4690-4-42

**Published:** 2007-06-18

**Authors:** Kuan-Teh Jeang

**Affiliations:** 1 National Institutes of Health, Bethesda, MD, USA

## Abstract

There is a natural tendency to judge a gift by the attractiveness of its wrapping. In some respect, this reflects current mores of measuring the gravitas of a scientific paper based on the journal cover in which the work appears. Most journals have an impact factor (IF) which some proudly display on their face page. Although historically journal IF has been a convenient quantitative shorthand, has its (mis)use contributed to inaccurate perceptions of the quality of scientific articles? Is now the time that equally convenient but more individually accurate metrics be adopted?

I surmise that a common question posed to an editor of a new journal is "What is your impact factor?" Based on my experience, in the majority of instances as the conversation evolves, it becomes evident that the questioner misunderstands what impact factor means. IF is a useful number. However, its limitations must be clearly recognized. Given the pervasive (if not obsessive) interest in IF, *Retrovirology*, as a new journal entering its fourth year of publication, has necessarily mined the citation databases and calculated IF numbers for 2005 (2.98) and 2006 (4.32) [1]. After having captured those numbers, it is perhaps instructive to consider some factual denotations and frequently misinterpreted connotations of IF. Indeed, as science and medicine march to a more personalized approach, one might further ask if it is time to embrace highly accessible technology in order to complement/supplant generic IF with individually precise citation metrics?

## Impact Factor --- what does it (not) say?

In the 1960s, the Institute for Scientific Information (ISI), a component of Thomson Scientific, a division of The Thomson Corporation (a publicly traded company engaged in financial services, healthcare sectors, law, science and technology research, and tax and accounting services) devised the "impact factor" (IF), a number developed for the purpose of comparing different journals [2].

IF gauges the standing of a journal for a specified year. Hence, IF can be viewed as the mean number of citations that occurred in a specific year to articles published in a journal during the two previous years. In common vernacular, IF reflects the number of times an "average article" in a journal has been cited per year in the two immediately preceding years. A poorly-understood nuance to this definition is that IF disproportionately favors citations made during the first two years subsequent to a paper's publication, and does not accurately capture the paper's "value" over a longer time. Hypothetically, let's consider two papers that receive the same total number of citations (e.g. 100 times) over a 10 year period. Paper A is cited 80 times in its first two immediate calendar years after publication, and then 20 times over the subsequent eight years. Paper B is cited 20 times over the first two years and 80 times over the next eight years. Paper A fits the profile of an average article published by the journal *"FlashyStuff"*; while paper B is a usual paper in the journal "*ReliablySolid*". Within the context of this example, *FlashyStuff *would sport an IF of 40 while *ReliablySolid *would have an IF of 10. Intriguingly, the rather impressive 4 fold difference in impact factor belies common sense --- that over ten years, a *FlashyStuff *article is cited no more frequently than a *ReliablySolid *paper (both exactly 100 times).

The above discussion briefly spotlights what IF in part does and does not convey. With that disclaimer, how is *Retrovirology *doing IF-wise as the journal enters its fourth year? Employing the algorithm that IF derives from the number of citations to a journal (by other ISI tracked journals) divided by the "citable items" published in the journal, in 2005 *Retrovirology *had a calculated IF of 2.98 based on ISI data. For 2006, *Retrovirology's *IF is calculated using the following parameters : N (numerator) = the number of times *Retrovirology *articles published in 2004 and 2005 were cited by other journals during the single 2006 year, and D (denominator) = the total number of citable *Retrovirology *articles published in years 2004 and 2005. *Retrovirology's *2006 IF is then N divided by D = 4.32.

**Figure 1:**
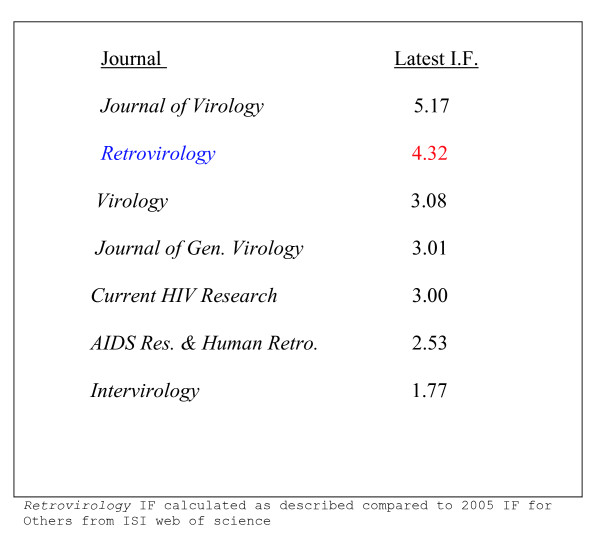
A comparison of *Retrovirology's *calculated 2006 impact factor with selected journals that publish retrovirus research papers. Values for other journals are from ISI journal citation report for 2005.

How does 4.32 stack up against numbers from other journals? Figure 1 compares *Retrovirology's *4.32 IF with recent IF numbers of other virology journals that frequently publish basic research on retroviruses. When measured against *Journal of Virology*, *Journal of General Virology*, *Virology*, *Current HIV Research*, *AIDS Research and Human Retroviruses*, and *Intervirology*, *Retrovirology*, the only Open Access publication amongst the seven, indeed rates very competitively (Fig. 1).

## Now for something completely different?

In the late 1970's when I began graduate school, large bulky word processing machines were just being invented, and small personal computers did not exist. This was a period when if one wished to learn what was being published, one had to reach for the weekly/monthly periodicals (that often meandered through the postal service sometimes, if it is a foreign journal, arriving months after publication) which were displayed on reading shelves in libraries. PubMed, other electronic databases, email, keyword e-alert, and instant table of contents notification were science fiction. In that era, it was laborious and time consuming to assess individually a journal's or a colleague's citation records. Hence, back then, judging a "book by its cover" or rating a paper based on the journal's IF would seem excusable simply because there was little other practical recourse.

In 2007, one can do much better. In the fifty years since the advent of IF, a couple of salient shortcomings to this index have been noted. First, an inherent quirk to IF definition allows the numerator to contain citations to "non-citable" items that are not counted in the denominator [3]. In general, "non-citable" items are the short newsy/opinion/commentary "front matter" pieces written by professional writers which appear frequently in many "high impact" journals. Citations to "front matter" pieces are tallied in the numerator for a journal's IF without a commensurate "penalty" added to the denominator. Hence, venues that publish numerous "non-citable" front matters have "inflated" IFs relative to counterparts that publish only "citable" articles (e.g. original research papers). Second, a journal's IF is a poor surrogate indicator of individual articles published in that journal. There is a statistical pattern to citations that on average 15% of the articles in a journal accounts for 50% of all citations to that journal, and the top 50% of articles in a journal garner 90% of citations to that journal [4]. Thus, a top 50% article can be cited 10 times more frequently than a bottom 50% article in the same journal [4]. Given the likelihood of a 10 fold difference in actual citations, why should colleagues assume that one *Cell/Science/Nature *paper has remotely the same value as another? Hence, even if one accepts citation frequency as a reflection of quality, there is little reason to adopt a journal's overall IF as a reliable touchstone for the gamut of papers published in that journal. The umbrella-like use of IF as a general quality tag seems all the more unnecessary since there are so many rapid and accessible options for tracking article-specific citations (e.g. Google Scholar, Scopus, and Web of Science [5]).

A couple of days ago, I read a remarkable news headline. "James Watson of DNA fame gets his own genome map". I recollected that when the first human genome sequencing was being done generic anonymity of that initial DNA was important. However, time has changed, and today James Watson (and you too) can have an individual genome sequenced rapidly and inexpensively. Is now not also the time that scientists should move to personalized measurements of citations? Aren't individual citation frequencies more thoughtful reflections of one's scientific corpus than the answer to the oft-bantered generic query "How many *Cell/Science/Nature *papers has he/she published?"

Today, one's individual citation frequency is easily accessible to all who have a few minutes to spend and internet access to databases. Google Scholar, Scopus, or Web of Science can each fully provide such information. Of the three, I found Scopus [6] to be the most user-friendly in its data organization and searchability. Hence, yesterday when I had a spare hour, I used Scopus to tabulate individual citation frequency and H index of 45 members of *Retrovirology's *editorial board (Table 1). {The H index is another way to quantify a scientist's quality and quantity of scholarly output [7,8]. This index attempts to combine and balance the effect of "quantity" (number of publications) and "quality" (citation rate) in a specific way.} I should point out that I did this data collection quickly (each person's numbers took no more than 1 minute), and as with all databases and human entries there can be errors (apologies to colleagues if I made mistakes with your numbers). Hence, please take table 1 to be illustrative rather than factually literal. Nevertheless, this elementary exercise echoes the words of a past US president, "You can run, but you cannot hide." Like it or not, use it or not, each author's personalized citation number and H index are there for all to compare.

**Table 1:** Citation frequency and H index for selected *Retrovirology *Editorial Board members (data collated on June 11, 2007 from Scopus).

**Title**	**Name**	**Role within** ***Retrovirology***	**Institution**	**City**	**Country**	**H index**	**Total times cited** ** since 1995**
Dr.	Kuan-Teh Jeang	Editor-in-Chief	NIH	Bethesda	USA	39	7724
Dr.	Monsef Benkirane	Editor	CNRS	Montpellier	France	15	1371
Dr.	Ben Berkhout	Editor	Academic Med. Ctr	Amsterdam	the Netherlands	32	4627
Dr.	Andrew Lever	Editor	Cambridge University	Cambridge	UK	16	1591
Dr.	Mark Wainberg	Editor	McGill University	Montreal	Canada	34	8457
Dr.	Masahiro Fujii	Editor	Niigata University	Niigata	Japan	18	1410
Dr.	Michael Lairmore	Editor	Ohio State University	Columbus	USA	17	1721
							
Dr.	Michael Bukrinsky	Ed Board	George Washington Univ	Washington DC	USA	21	4247
Dr.	Dong-yan Jin	Ed Board	Hong Kong U	Hong Kong	China	20	1896
Dr.	Klaus Strebel	Ed Board	NIH	Bethesda	USA	21	3218
Dr.	Tom J. Hope	Ed Board	U. Illinois	Chicago	USA	23	3609
Dr.	Serge Benichou	Ed Board	Cochin Institute	Paris	France	20	1466
Dr.	Stephane Emiliani	Ed Board	Cochin Institute	Paris	France	15	1506
Dr.	Olivier Bensaude	Ed Board	INSERM	Paris	France	20	2046
Dr.	Mauro Giacca	Ed Board	Int. Ctr. Genetics	Trieste	Italy	33	4577
Dr.	Olivier Schwartz	Ed Board	Institut Pasteur	Paris	France	26	3865
Dr.	Leonid Margolis	Ed Board	National Inst Child Health	Bethesda	USA	17	1394
Dr.	Fatah Kashanchi	Ed Board	George Washington U.	Washington DC	USA	24	2071
Dr.	Masao Matsuoka	Ed Board	Kyoto University	Kyoto	Japan	19	1554
Dr.	Naoki Mori	Ed Board	University of the Ryukyus	Okinawa	Japan	24	1727
Dr.	Chou-Zen Giam	Ed Board	Uniform Services Med Sch	Bethesda	USA	14	1288
Dr.	David Derse	Ed Board	NCI	Frederick	USA	12	1488
Dr.	Tatsuo Shioda	Ed Board	Osaka Univ	Osaka	Japan	21	1605
Dr.	John Semmes	Ed Board	Eastern Virginia Med College	Norfolk	USA	23	2230
Dr.	John Hiscott	Ed Board	McGill Univ.	Montreal	Canada	37	6134
Dr.	Fabrizio Mammano	Ed Board	INSERM	Paris	France	14	1485
Dr.	James K. Hildreth	Ed Board	Meharry Univ.	Nashville	USA	23	2305
Dr.	Finn Skou Pedersen	Ed Board	University of Aarhus	Aarhus	Denmark	17	1169
Dr.	Janice Clements	Ed Board	Johns Hopkins Med School	Baltimore	USA	21	2879
Dr.	Tahir A. Rizvi	Ed Board	United Arab Emirates Univ.	Al Ain	UAE	14	1133
Dr.	Chris Aiken	Ed Board	Vanderbilt University	Nashville	USA	15	1593
Dr.	Neil Almond	Ed Board	NIBSC	Potters Bar	UK	12	1132
Dr.	Stephen P. Goff	Ed Board	Columbia University	New York	USA	33	8325
Dr.	Johnson Mak	Ed Board	Burnet Inst. Med. Research	Victoria	Australia	14	1094
Dr.	Christine Kozak	Ed Board	NIH	Bethesda	USA	27	6967
Dr.	Greg Towers	Ed Board	Univ. College	London	UK	14	1047
Dr.	Graham Taylor	Ed Board	Imperial College	London	UK	20	1710
Dr.	Eric Cohen	Ed Board	Univ. Montreal	Montreal	Canada	31	5050
Dr.	William Hall	Ed Board	University College Dublin	Dublin	Ireland	16	1293
Dr.	Warner Greene	Ed Board	UCSF	San Francisco	USA	35	8114
Dr.	Jean-luc Darlix	Ed Board	U. Lyon	Lyon	France	27	4816
Dr.	Axel Rethwilm	Ed Board	U. Wuerzburg	Wuerzburg	Germany	21	1784
Dr.	Eric Freed	Ed Board	NCI	Frederick	USA	27	3495
Dr.	Toshiki Watanabe	Ed Board	Univ. of Tokyo	Tokyo	Japan	18	1276
Dr.	Mari Kannagi	Ed Board	Tokyo Med and Dental U	Tokyo	Japan	14	1189

## Gilt by association?

How then should one choose where to publish one's manuscript? It has been raised that many scientists employ the "gilt by association" [9] approach, first sending their papers to high-visibility, high-IF journals, perhaps hoping that the "free ride" hypothesis works [4] and some of a journal's sheen would direct attention to and rub off on the work. However, there is no factual evidence that publishing a paper in a highly touted journal adds "free citations" to a paper other than those achieved by its content [4]. Indeed, this point seems to make intuitive sense. For example, a paper is the same paper if it were initially declined at *Cell *and then published in the *Journal of Virology *than if the paper were quickly accepted at *Cell *and did not have to make the rounds to the *Journal of Virology*. If the paper remains the same, should one sequence of events confer higher inherent quality to the same paper over another?

In the past, to support the interest of equal access to knowledge by scientists and students in developing economies who cannot afford subscription-based journals, I have argued that we have a responsibility to support Open Access publishing [10]. From a principled point of view, not to do so is poorly defensible. On the other hand, if one formulates decisions using a self-interest citation frequency driven perspective, evidence similarly supports that in head-to-head comparisons Open Access articles are cited more frequently than non-Open Access counterparts [11]. In today's publishing world, there are important roles to be played by both subscription and Open Access journals. However, as Open Access journals ascend in quality and visibility and globalization brings us closer to previously distant strangers, scientists confident in the inherent content and value of their papers might ask if they can tolerate the "guilt associated with not supporting egalitarian access"?

The above is a difficult question that each individual has to ponder. I am grateful for the answer that many authors and editorial board members of *Retrovirology *have provided to this question.
